# Editorial: Strain-specific probiotics: enhancing children's health through targeted clinical research

**DOI:** 10.3389/fnut.2026.1888544

**Published:** 2026-06-16

**Authors:** Ke Chen

**Affiliations:** Department of Clinical Nutrition, Chengdu Women's & Children's Central Hospital, School of Medicine, University of Electronic Science and Technology of China, Chengdu, China

**Keywords:** gastrointestinal microbiome, immunomodulation, child health, precision medicine, probiotics

The human microbiota is now widely recognized as a critical determinant of pediatric health. During infancy and childhood, the establishment and maturation of the gut microbiome shape immune education, gastrointestinal function, metabolic programming, and neurodevelopment. Disruptions in microbial colonization during these early stages have been linked to recurrent infections, allergic diseases, obesity, neurodevelopmental conditions, and inflammatory disorders. Against this background, probiotics have attracted considerable scientific and clinical interest as potentially safe, microbiota-targeted interventions.

Yet a major challenge persists. Clinical outcomes remain highly heterogeneous, and evidence increasingly shows that probiotic effects are strain-specific. Benefits observed with one strain cannot be generalized across species, or even across different strains within the same species. Efficacy depends on strain characteristics, dosage, timing of administration, disease phenotype, host age, baseline microbiota composition, and environmental context.

The Research Topic “*Strain-specific probiotics: enhancing children's health through targeted clinical research*” was developed to address these challenges and to promote evidence-based pediatric probiotic research grounded in precise strain identification and rigorous clinical validation. This collection brought together original clinical studies, randomized controlled trials, systematic reviews, umbrella reviews, and mechanistic analyses exploring the role of defined probiotic strains in improving child health outcomes. It emphasized translational relevance, integrating clinical efficacy with microbiota analyses, immune biomarkers, and mechanistic insights.

The 23 articles in this Research Topic illustrate the rapidly evolving landscape of pediatric microbiota-targeted interventions. The contributions cover a broad spectrum of conditions, including respiratory infections, gastrointestinal diseases, allergic disorders, obesity, neurodevelopmental conditions, environmental toxin exposure, and rare genetic syndromes. Although diverse in focus, the studies consistently reinforce several key principles: probiotics are not interchangeable, mechanistic precision matters, and pediatric probiotic interventions require disease-specific and strain-specific evaluation.

## Immune modulation and infectious disease

A major theme emerging from the collection is the role of probiotics in modulating immune function and reducing infections in children. Several randomized controlled studies investigated *Bifidobacterium longum* subsp. *infantis* YLGB-1496 in young children, demonstrating beneficial effects on common pediatric illnesses, immune regulation, and gut microbiota composition [Zhang, Chen, et al. (a), Uma Mageswary, Li, et al.]. Improvements in respiratory symptoms, gastrointestinal health, and immune-related biomarkers suggest that early microbiota modulation may strengthen host defense during critical periods of immune maturation.

These findings carry particular weight because early childhood represents a window of developmental vulnerability. Environmental exposures, feeding practices, antibiotic use, and microbial colonization patterns during this period may exert long-term health effects. The observed benefits of targeted bifidobacterial supplementation support the hypothesis that selective microbiota interventions can help optimize immune resilience.

Respiratory health represented another important area of investigation. Several studies and reviews explored the relationship between probiotics and pediatric respiratory diseases, including recurrent upper respiratory tract infections (Uma Mageswary, Lan, et al., Chen, Jin, et al.) (1). One randomized controlled trial evaluating probiotic supplementation in infants reported improvements in both respiratory and gastrointestinal outcomes, accompanied by favorable shifts in gut microbial composition and immune parameters (Uma Mageswary, Lan, et al.).

Narrative reviews also summarized accumulating evidence supporting strain-specific probiotics, particularly *Lacticaseibacillus rhamnosus* GG, in respiratory disease prevention (1, 2). These studies contribute to the growing understanding of the gut–lung axis, the bidirectional network linking intestinal microbiota with pulmonary immune responses.

## Allergic disease prevention

The collection made important contributions to allergic disease prevention and immune modulation. Allergic disorders continue to increase among children globally, and microbiota dysbiosis has been proposed as a major contributing factor. One clinical study investigated *Bifidobacterium lactis* BLa80 supplementation in reducing eczema incidence and respiratory infections in early childhood, demonstrating beneficial effects on microbiota balance and immune regulation (Chen, Jin, et al.).

Systematic and umbrella reviews evaluated current evidence regarding probiotics in atopic dermatitis prevention and management (Zhong et al.) (3). While some strains showed encouraging preventive effects, the reviews emphasized substantial heterogeneity in study design, probiotic formulation, timing of intervention, and clinical endpoints.

## Gastrointestinal and systemic health

Gastrointestinal diseases remain among the most established applications of probiotics in pediatric medicine, and multiple articles expanded the evidence base. Acute gastroenteritis, antibiotic-associated diarrhea, and intestinal dysbiosis continue to represent major global health burdens in infants and young children.

One systematic review evaluated the combined use of *Saccharomyces boulardii* CNCM I-745 and smectite in children with acute gastroenteritis, reporting favorable effects on symptom duration and recovery (Li and McFarland). Another multicenter clinical study explored probiotic co-treatment for antibiotic-associated diarrhea, demonstrating benefits in symptom control and treatment tolerance (Liu et al.). Additional reviews summarized the role of probiotics in pediatric gastrointestinal disorders and necrotizing enterocolitis prevention (4–6).

Several articles moved beyond traditional gastrointestinal endpoints to explore broader systemic implications of microbiota modulation. Researchers increasingly recognize that gut microbial communities influence not only digestion and immunity, but also metabolic, endocrine, and neurobehavioral pathways.

## Neurodevelopment and metabolism

A particularly noteworthy aspect of this Research Topic was the inclusion of studies investigating the microbiota–gut–brain axis and neurodevelopmental disorders. Reviews focusing on autism spectrum disorder summarized recent advances in dietary interventions, microbiota modulation, and probiotic strategies aimed at improving gastrointestinal and behavioral symptoms (Fang et al.). Alterations in microbial diversity, intestinal permeability, microbial metabolites, and immune activation may contribute to autism-related pathophysiology.

Another review explored the association between trans fatty acid exposure, gut microbiota dysbiosis, inflammation, and attention-deficit/hyperactivity disorder (ADHD) (He et al.). By integrating nutritional, microbial, and neurodevelopmental perspectives, these contributions reflect the increasingly interdisciplinary nature of pediatric microbiome research.

The Research Topic also addressed obesity and metabolic dysfunction in children. Several articles discussed how probiotic interventions may influence metabolic homeostasis, adiposity, inflammation, and intestinal barrier integrity (7).

## Rare and specialized clinical contexts

Beyond common pediatric conditions, the collection included studies in rare or specialized clinical settings. One systematic review explored microbiota-targeted interventions in children with Prader–Willi syndrome, highlighting emerging evidence that microbiota modulation may provide adjunctive therapeutic benefits in this challenging population (Toh et al.).

Another innovative contribution examined *Lactiplantibacillus plantarum* DSM 33464 in children with elevated blood lead levels. This randomized placebo-controlled trial suggested that probiotic supplementation may have detoxification-related potential through modulation of intestinal barrier function, microbial metabolism, and heavy metal handling (Ji et al.).

## Methodological advances and future directions

Several articles explored methodological and conceptual issues relevant to pediatric probiotic research, emphasizing the importance of strain characterization, genomic identification, viability assessment, dosage standardization, and long-term safety evaluation [Zhang, Chen, et al. (b)] (8–10). As probiotic research matures, precise taxonomic identification and mechanistic validation are becoming essential components of high-quality translational studies.

Another important theme was the integration of multi-omics technologies. Many studies incorporated microbiome sequencing, metabolomic analyses, cytokine profiling, and immune biomarker assessment, allowing researchers to move beyond descriptive clinical outcomes and identify functional pathways linking probiotic interventions with host physiology.

The collection also underscores the importance of developmental timing. Infancy and early childhood are periods of rapid microbial and immune maturation, during which interventions may exert disproportionately large biological effects. Several studies suggested that early-life probiotic supplementation may influence long-term immune tolerance, allergic susceptibility, and metabolic programming.

## Ongoing challenges

Despite encouraging findings, the collection highlights persistent challenges. First, substantial heterogeneity remains across clinical trials regarding strain selection, dosing regimens, intervention duration, and outcome assessment. This variability complicates meta-analyses and limits generalizability.

Second, while many studies demonstrate statistically significant improvements in surrogate biomarkers or symptom scores, clinically meaningful endpoints and long-term follow-up data remain limited. Future research should prioritize standardized outcome measures, multicenter collaborations, and adequately powered randomized controlled trials.

Third, host-related factors—including baseline microbiota composition, dietary patterns, genetics, environmental exposures, delivery mode, breastfeeding status, and antibiotic history—may substantially influence probiotic responsiveness. Precision probiotic approaches tailored to individual microbial and immunological profiles may therefore represent an important future direction.

Finally, regulatory and translational challenges remain. Although probiotics are widely marketed globally, the scientific rigor supporting different formulations varies considerably. This Research Topic reinforces the necessity of evidence-based clinical recommendations grounded in strain-specific data rather than generalized claims.

## Conclusion

The articles included in this Research Topic illustrate the progress occurring in pediatric microbiome and probiotic research. They highlight how strain-specific probiotic interventions may influence gastrointestinal, respiratory, allergic, metabolic, and neurodevelopmental health outcomes through complex interactions involving the microbiota, immune system, metabolism, and host physiology.

At the same time, the collection emphasizes that probiotics should not be viewed as universally interchangeable therapeutic agents. Future progress will depend on rigorous strain-specific validation, mechanistic understanding, and carefully designed pediatric clinical trials.

As microbiome science evolves, pediatric probiotic research is moving toward precision medicine approaches that integrate microbial ecology, immunology, nutrition, and systems biology. The studies gathered here contribute meaningfully to this transition and provide a foundation for future investigation.

We thank all authors, reviewers, editors, and researchers who contributed to this Research Topic. Their work advances our understanding of microbiota-targeted therapies and supports the development of safer, more effective, and scientifically grounded probiotic interventions for children.

## References

[1] Xavier-Santos D Scharlack NK de Lima Pena F Antunes AEC. Effects of *Lacticaseibacillus rhamnosus* GG supplementation, via food and non-food matrices, on children's health promotion: a scoping review. Food Res Int. (2022) 158:111518. doi: 10.1016/j.foodres.2022.111518 35840226

[2] Szajewska H Kolodziej M. Prophylactic use of probiotics for gastrointestinal disorders in children. Lancet Child Adolesc Health. (2019) 3:655–62. doi: 10.1016/S2352-4642(19)30182-8 31279590

[3] Tan W Zhang Q Liu W Yi J Qiu Z. Lactobacillus rhamnosus GG for cow's milk allergy in children: a systematic review and meta-analysis. Front Pediatr. (2021) 9:791585. doi: 10.3389/fped.2021.727127 34746056 PMC8569903

[4] Capozza M Panza R Laforgia N Baldassarre ME. Probiotics and functional gastrointestinal disorders in pediatric age: a narrative review. Front Pediatr. (2022) 10:805466. doi: 10.3389/fped.2022.805466 35252059 PMC8888932

[5] Priyadarshi A Lowe G Saddi V Trivedi A Luig M Tracy M. Clinical outcomes of single vs. two-strain probiotic prophylaxis for prevention of necrotizing enterocolitis in preterm infants. Front Pediatr. (2021) 9:729535. doi: 10.3389/fped.2021.729535 34527647 PMC8435710

[6] Tierney BT Versalovic J Fasano A Petrosino JF Chumpitazi BP Mayer EA. Functional response to a microbial synbiotic in the gastrointestinal system of children: a randomized clinical trial. Pediatr Res. (2023) 93:2005–13. doi: 10.1038/s41390-022-02289-0 36319696 PMC10313516

[7] Petraroli M Castellone E Patianna V Esposito S. Gut microbiota and obesity in adults and children: the state of the art. Front Pediatr. (2021) 9:657020. doi: 10.3389/fped.2021.657020 33816411 PMC8017119

[8] Tremblay A Bronner S Binda S. Review and perspectives on Bifidobacterium lactis for infants' and children's health. Microorganisms. (2023) 11:2501. doi: 10.3390/microorganisms11102501 37894159 PMC10609373

[9] Daglia M Drago L Ullah H Di Minno A Brindisi G Brunese FP . Effects of the supplementation of single and multi-strain probiotics, alone or in combination with other treatments, on asthma in children: a systematic review of the randomized, placebo-controlled clinical studies. J Funct Foods. (2024) 123:106599. doi: 10.1016/j.jff.2024.106599

[10] Nguyen M Holdbrooks H Mishra P Abrantes M Eskew S Garma M . Impact of probiotic B. infantis EVC001 feeding in premature infants on the gut microbiome, nosocomially acquired antibiotic resistance, and enteric inflammation. Front Pediatr. (2021) 9:618009. doi: 10.3389/fped.2021.618009 33665175 PMC7921802

